# De-duplicating patient records from three independent data sources reveals the incidence of rare neuromuscular disorders in Germany

**DOI:** 10.1186/s13023-019-1125-2

**Published:** 2019-06-24

**Authors:** Kirsten König, Astrid Pechmann, Simone Thiele, Maggie C. Walter, David Schorling, Adrian Tassoni, Hanns Lochmüller, Clemens Müller-Reible, Janbernd Kirschner

**Affiliations:** 1 Department of Neuropediatrics and Muscle Disorders, Medical Center- University of Freiburg, Faculty of Medicine, University of Freiburg, Freiburg, Germany; 2 Clinical Trials Unit, Medical Center – University of Freiburg, Faculty of Medicine, University of Freiburg, Freiburg, Germany; 3 Department of Neurology, Friedrich-Baur-Institute, Ludwig-Maximilians-University of Munich, Munich, Germany; 4 Centro Nacional de Análisis Genómico (CNAG-CRG), Center for Genomic Regulation, Barcelona Institute of Science and Technology (BIST), Barcelona, Catalonia Spain; 5 Children’s Hospital of Eastern Ontario Research Institute, University of Ottawa, Ottawa, Canada; 6 Division of Neurology, Department of Medicine, The Ottawa Hospital, Ottawa, Canada; 7 Institute of Human Genetics, University of Würzburg, Würzburg, Germany; 8 Department of Neuropediatrics, University Hospital Bonn, Bonn, Germany

**Keywords:** Incidence, Neuromuscular disease, Spinal muscular atrophy, Duchenne muscular dystrophy

## Abstract

**Background:**

Estimation of incidence in rare diseases is often challenging due to unspecific and incomplete coding and recording systems. Patient- and health care provider-driven data collections are held with different organizations behind firewalls to protect the privacy of patients. They tend to be fragmented, incomplete and their aggregation leads to further inaccuracies, as the duplicated records cannot easily be identified. We here report about a novel approach to evaluate the incidences of Duchenne muscular dystrophy (DMD) and spinal muscular atrophy (SMA) in Germany.

**Methods:**

We performed a retrospective epidemiological study collecting data from patients with dystrophinopathies (DMD and Becker muscular dystrophy) and SMA born between 1995 and 2018. We invited all neuromuscular centers, genetic institutes and the patient registries for DMD and SMA in Germany to participate in the data collection. A novel web-based application for data entry was developed converting patient identifying information into a hash code. Duplicate entries were reliably allocated to the distinct patient.

**Results:**

We collected 5409 data entries in our web-based database representing 1955 distinct patients with dystrophinopathies and 1287 patients with SMA. 55.0% of distinct patients were found in one of the 3 data sources only, while 32.0% were found in 2, and 13.0% in all 3 data sources. The highest number of SMA patients was reported by genetic testing laboratories, while for DMD the highest number was reported by the clinical specialist centers. After the removal of duplicate records, the highest yearly incidence for DMD was calculated as 2.57:10,000 in 2001 and the highest incidence for SMA as 1.36:10,000 in 2014.

**Conclusion:**

With our novel approach (compliant with data protection regulations), we were able to identify unique patient records and estimate the incidence of DMD and SMA in Germany combining and de-duplicating data from patient registries, genetic institutes, and clinical care centers. Although we combined three different data sources, an unknown number of patients might not have been reported by any of these sources. Therefore, our results reflect the minimal incidence of these diseases.

**Electronic supplementary material:**

The online version of this article (10.1186/s13023-019-1125-2) contains supplementary material, which is available to authorized users.

## Background

Very few countries such as Denmark capture all patients with rare, neuromuscular disorders in a centralized database through mandatory reporting via the health care system. Therefore, the data of all Danish patients is known and allow for targeted care provision and planning [1]. In contrast, in most other countries, health care is organized by regions or provinces, and data capture for rare disease patients is scattered, fragmented and voluntary. Movement of patients between regions and health care providers, and data capture through different organizations and for different purposes lead to a duplication of records for the same individual, which cannot be easily corrected for if the personal information of the individual is protected in accordance with data protection regulation. Furthermore, the coding systems may not be compatible between the different data sources, potentially compromising the validity of any conclusions drawn from combining datasets even if de-duplication can be achieved. The International Rare Disease Research Consortium (IRDiRC) has convened a task force that developed principles and concepts for privacy protecting record linkage (PPRL) for rare diseases [2], similar to what has been applied in the cancer field (EUPID) [3]. Technically, these systems rely in part on hash codes that allow de-duplication of records while not revealing personal identifiable information (PII) of the individual. So far, they have not been utilized for rare neuromuscular disease on a larger scale.

Assessing the incidence of rare diseases is challenging. Due to unspecific coding systems (e.g. the ICD-10 system), health system data are not suitable to assess the incidence or prevalence of a given rare disease in most countries. Patient registries are often used to estimate patient numbers and to evaluate the care for patients with a given rare disease. However, as registering is voluntary it is clear that patient registries cover only a part of the population and are also associated with a bias towards more active and better cared for patients [4]. However, without reliable data on incidence or prevalence, it is extremely difficult to evaluate, plan and improve health care for people with rare diseases. Several data sources have been used to estimate the incidence and prevalence of DMD and SMA including patient registries [1], reports from neuromuscular centers [5] or reports from genetic institutes [6]. For Germany, none of these sources provides full coverage and so far, it was not possible to identify to what extent distinct or identical patients are reported by the different sources. To overcome this problem we developed a web-based database with onsite generation of unique hash codes that allow identifying patient duplicates between the different sources.

## Methods

We conducted a retrospective epidemiological study to determine the incidences of DMD and SMA in Germany, respectively. Data was collected from neuromuscular centers, genetic institutes and the German patient registries. Seventy two neuromuscular centers were identified by the Care and Trail Site Registry (CTSR) [5] and the German patient organization for neuromuscular disorders (Deutsche Gesellschaft für Muskelkranke e.V. (DGM)). Genetic institutes were identified using publically available information as well as expert input and validation using the following sources: the Orphanet database of diagnostic laboratories, the German society of human genetics (Deutsche Gesellschaft für Humangenetik e.V.), and the professional association of German human geneticists (Berufsverband Deutscher Humangenetiker e.V.). Neuromuscular centers and genetic institutes were initially invited by mail to participate in our data collection. Additionally, the German patient registries for DMD or SMA (www.dmd-register.de and www.sma-register.de) hosted by the Friedrich-Baur-Institute of the University of Munich provided data of registered patients [7, 8]. Data was collected from May 2017 to August 2018.

For data collection, a password-protected web-based database was developed. The hash code was created from the birth date and patient’s initials in the local browser directly after data entry. Therefore, only the hash code and no PII was transferred to the server. The hash code and the entered data was stored on a dedicated server at the University of Cologne. Since the hash code was unique to each patient, it was possible to identify duplicate entries between the different data sources. A hash code by definition cannot be decrypted. To inhibit possible resolving of the hash code to the original data by brute force attacks, a specially secure and slow algorithm was used to create the hash code (Bcrypt, https://github.com/fpirsch/twin-bcrypt). The algorithm was based on a Blowfish cipher and implemented in JavaScript. The hash code system was tested in various browsers to verify reliability and collision resistance of the algorithm. To secure data protection, data extracts from the database were limited to aggregated forms with a minimum group size of five. With this approach, patients’ consent was not required.

As available information is different between care centers, genetic institutes and patient registries, the requested dataset was slightly adjusted. The web-based application provided a short questionnaire for data entry (for details see additional file 1). We asked all data sources to enter data of patients with either dystrophinopathies (including DMD or Becker muscular dystrophy (BMD)) or SMA born between 1995 and 2018. In case of discrepancies between the different data sources regarding the classification of SMA types or differentiation between DMD or BMD, the diagnosis form neuromuscular centers was used for the final classification of the disease type. Patients with discrepant entries form two different neuromuscular centers were not allocated to a subgroup. To calculate incidences, we used data from the German federal statistical office (www.destatis.de).

Descriptive data analyses were performed by calculation of absolute frequencies and percentages. Diagrams using ellipses were developed with eulerAPE [9]. The data protection officer and the Ethics committee at the Medical Center – University of Freiburg, approved this project.

## Results

In total, 32 neuromuscular centers and 12 genetic institutes participated in our data collection.

**Fig. 1:**
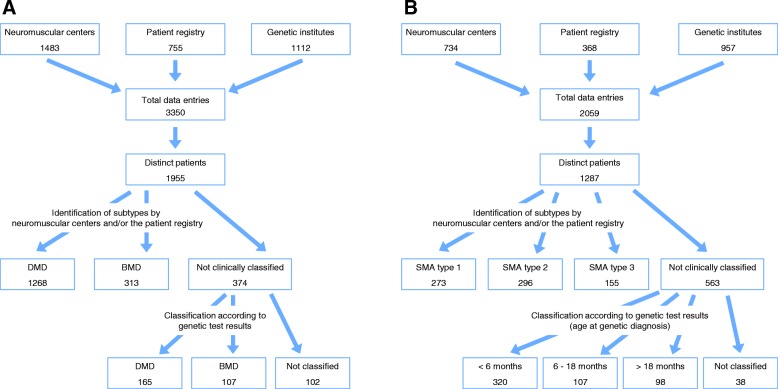
Presentation of all data entries by neuromuscular centers, genetic institutes and the patient registry, the identification of distinct patients and the allocation to different subtypes of dystrophinopathy (**a**) or SMA (**b**)

**Fig. 2:**
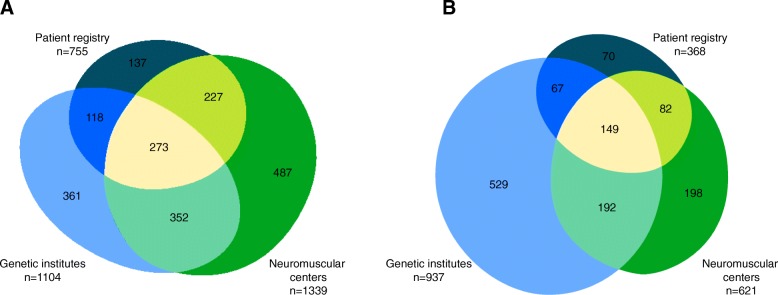
Data entries of distinct patients with **a** dystrophinopathies or **b** SMA subdivided based on the different data sources. Data entered only by genetic institutes is displayed in light blue, data from neuromuscular centers in green and data from the patient registry in teal

We counted in total 3350 data entries on patients with dystrophinopathies from neuromuscular centers, the patient registry and genetic institutes. Among these, we identified 1955 distinct patients: 985 patients (50.4%) were found in one of the 3 data sources only, while 697 (35.7%) were found in 2 of the data sources. The overlap between all three data sources was about 273 distinct patients (14.0%). Of all patients with dystrophinopathies, 1433 (73.3%) were classified as DMD and 420 (21.5%) as BMD. In 102 patients (5.2%) classification to these subgroups was not possible. We further collected 2059 data entries on patients with SMA representing 1287 distinct patients: 797 patients (61.9%) were found in one of the 3 data sources only, while 341 (26.5%) were found in 2 and 149 (11.6%) in all 3 data sources. Data on subtypes of SMA was collected only from neuromuscular centers and the patient registry (in total 758 patients). Of these, 273 patients (36.0%) were classified as SMA type 1, 296 (39.1%) as SMA type 2 and 155 (20.4%) as SMA type 3. In 34 patients of these patients (4.5%), classification to a subtype of SMA was not possible. Further, 529 patients (41.1%) were only reported by a genetic institute without information on the subtype of SMA. Of these patients, 320 (60.5%) patients were genetically diagnosed within the first 6 months of life, 107 patients (22.2%) between the age of 6 and 18 months, and 98 patients (18.5%) after the age of 18 months. Highlighting the subgroup of SMA patients entered by a genetic institute and a neuromuscular center, 94.6% of patients diagnosed within the first 6 months of life were clinically classified as SMA type 1 by the neuromuscular center. The respective data is provided in the additional file 2. Data for patients with dystrophinopathies and SMA is summarized in Figs. 1 and 2.

With our approach, the highest incidence for DMD was 2.57:10,000 in 2001. In SMA, the highest incidence was 1.36:10,000 in 2014. A table with all incidences for DMD and SMA based on the updated natality rate in Germany is provided in the additional file 3.

**Fig. 3:**
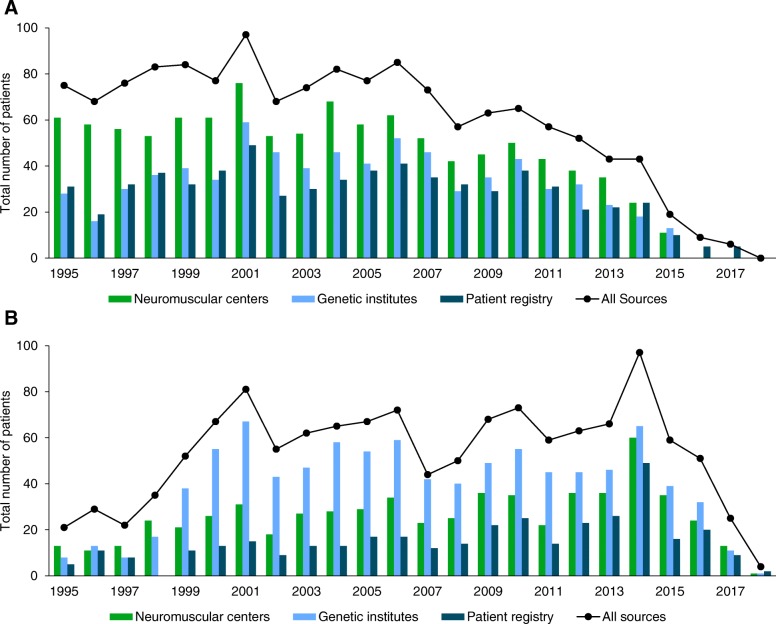
Data stratified by year of birth and the data source. **a** patients with DMD; **b** patients with SMA

We observed that in patients with DMD born before 2000, the majority of patients were identified by neuromuscular centers. In contrast, genetic institutes reported most of the patients with SMA especially in those born before 2012. The proportion of patients entered by the patient registry was higher in DMD than in SMA but remained underrepresented (see Fig. 3).

Data on the regional distribution of patients with dystrophinopathies or SMA is provided in the additional file 4.

## Discussion

With the aim to better understand the coverage and overlap between different data sources and to provide estimates for the incidences of DMD and SMA, respectively, we developed a novel web-based hash-coding database to collect data from neuromuscular centers, genetic institutes and the respective patient registries in Germany.

Many previous studies either referred to genetic or clinical diagnosis [10, 11] and did not combine different sources of information. In our cohort, a relevant number of patients was only identified by one of the data sources. For example, 24.9% of patients with dystrophinopathies were only reported by a neuromuscular center and, 41.1% of SMA patients were only identified by a genetic institute. Thus, our data confirm that none of the three data sources has sufficient coverage to provide reliable incidence estimates by itself. Further, previous studies discussed an unknown overlap between different sources not being able to identify distinct patients in case of duplicate entries [6]. With our approach, PII was converted into a hash code when entering the data allowing us to collect data without patients’ informed consent but still fulfilling the guidelines of General Data Protection Regulation (GDPR). The hash code reliably allocated duplicate entries to the distinct patients and thus, prevented an overestimation of incidences. Although we combined three different data sources, an unknown number of patients might not have been reported by any of these sources. Therefore, our results reflect the minimal incidence of these diseases.

Only neuromuscular centers and the patient registry provided data on the clinical classification of SMA types. In our cohort, 36.0% of SMA patients were clinically diagnosed as SMA type 1. The high percentage of SMA patients only reported by genetic institutes compared to patients with dystrophinopathies (see Fig. 2) may be due to the high early mortality of the disease. Especially, many patients with SMA type 1 have died within the first years of life and are therefore not reported by the patient registry or the neuromuscular centers. Most of the patients only reported by a genetic institute were genetically diagnosed within the first 6 months of life corresponding most likely to the clinical course of SMA type 1. Thus, it is conceivable, that the actual percentage of SMA type 1 in our cohort might probably be higher.

In both diseases, only a small percentage of patients were reported by the patient registry. Patient registries play an important role in neuromuscular diseases, particularly for the recruitment and planning of clinical trials. Furthermore, data from patient registries enable studies on disease epidemiology, natural history, functional outcomes or real world evaluation of drug efficacy and post-marketing drug surveillance and thus to improve care of these patients [12, 13]. Additionally, patient registries make data on novel treatment options, current clinical trials and research results available to participating patients and families [12]. We observed that only 38.6% of patients with DMD and 28.7% of patients with SMA participated in the respective patient registry. Patients who are regularly followed by a neuromuscular center are supposed to be better informed and accordingly more likely to participate in a patient registry. In our cohort, about two-thirds of patients participating in the patient registry were regularly under care in a neuromuscular center (66.2% in DMD and 62.8% in SMA). Nevertheless, information about patient registries provided by treating physicians does not seem to be sufficient to achieve a better participation of patients.

Our results show differences in the regional distribution of patients with dystrophinopathies and SMA per number of inhabitants. On the one hand, an underreporting in certain regions might cause these differences possibly due to poorer access to specialized neuromuscular centers. On the other hand, a different distribution of age groups in the population might have an influence on our results as we only collected data from patients born after 1995.

The main limitation of our approach evaluating the incidences of DMD and SMA in Germany was restricted data due to GDPR. Without patients’ informed consent, we were not allowed to collect any PII such as the exact date of genetic diagnosis or the birth date. Data extracts from the database were limited to aggregated forms with a minimum group size of five so that we might have missed data entries within our subgroup data analyses. We were not able to evaluate the prevalence of DMD and SMA not having exact data on the vital status of patients in our cohort. Life expectancy in these diseases is changing due to innovative treatments and technical advancements [14, 15]. Therefore, it is not possible to estimate life expectancy in these patients for a reliable calculation of prevalence.

However, using two rare, neuromuscular disorders, our study provides proof-of-principle that a combination of records from multiple data sources and de-duplication of records in a large, federated health care environment is feasible, while protecting privacy in line with regulations. The technological solutions used might be part of broader, more sustainable efforts to establish privacy-protecting record linkage for rare diseases as envisaged by IRDiRC [16] and piloted by RD-Connect [17].

## Conclusion

With our novel approach, we could evaluate the coverage and overlap of different and scattered data sources, and provide more reliable estimates of the minimal incidences of DMD and SMA in Germany. These findings will be important for planning further research and care for patients with neuromuscular diseases in Germany.

## Additional files

Additional file 1: Data provided by the different data sources. Table showing the differences between datasets requested from neuromuscular centers, genetic institutes and patient registries. (PDF 61 kb)

Additional file 2: Comparison of data regarding age at genetic diagnosis and SMA type. Table with data of patients entered by a genetic institute and a neuromuscular center comparing age at genetic diagnosis and clinical classification of SMA type. (PDF 10 kb)

Additional file 3: Incidences based on the updated natality rate in Germany for DMD and SMA. Table showing all incidences for DMD and SMA based on the updated natality rate in Germany. (PDF 100 kb)

Additional file 4: Distinct patients per 100,000 inhabitants allocated to the first digit of the postal code with dystrophinopathies or SMA. Map of Germany presenting data on the regional distribution of patients with dystrophinopathies or SMA. (PDF 240 kb)

## Data Availability

All data generated or analysed during this study are included in this published article [and its supplementary information files].

## Abbreviations

BMD: Becker muscular dystrophy
CTSR: Care and Trail Site Registry
DGM: Deutsche gesellschaft für muskelkranke
DMD: Duchenne muscular dystrophy
EUPID: European Unified Patient Identity Management
GDPR: General Data Protection Regulation
IRDiRC: International Rare Disease Research Consortium
PII: Personal identifiable information
PPRL: Privacy protecting record linkage
SMA: Spinal muscular atrophy
