# Supplementary material for: De-duplicating patient records from three independent data sources reveals the incidence of rare neuromuscular disorders in Germany
Source: Orphanet J Rare Dis. 2019 Jun 24;14:152. doi: 10.1186/s13023-019-1125-2 (PMC6591958; doi:10.1186/s13023-019-1125-2)
Supplement: Supplementary file 1 — Data provided by the different data sources. Table showing the differences between datasets requested from neuromuscular centers, genetic institutes and patient registries. (PDF 61 kb) [file 13023_2019_1125_MOESM1_ESM.pdf]

Data provided by the different data sources

	Neuromuscular centers	Patient registry	Genetic institutes
Year of birth	x	x	x
Vital status of the patient	x	x	
Diagnosis of the disease	x	x	x
Clinical classification of SMA types and dystrophinopathies	x	x	
Two first digits of the postal code of the patients' residence	x	x	x
Age at diagnosis (only in SMA)			x
Genetic testing approach (only in dystrophinopathies)			x
