# Supplementary material for: De-duplicating patient records from three independent data sources reveals the incidence of rare neuromuscular disorders in Germany
Source: Orphanet J Rare Dis. 2019 Jun 24;14:152. doi: 10.1186/s13023-019-1125-2 (PMC6591958; doi:10.1186/s13023-019-1125-2)
Supplement: Supplementary file 2 — Comparison of data regarding age at genetic diagnosis and SMA type. Table with data of patients entered by a genetic institute and a neuromuscular center comparing age at genetic diagnosis and clinical classification of SMA type. (PDF 10 kb) [file 13023_2019_1125_MOESM2_ESM.pdf]

Comparison of data regarding age at genetic diagnosis and SMA type

Age at genetic diagnosis / SMA type	Within the first 6 months of life	Between the age of 6 and 18 months	After the age of 18 months	Total
SMA type 1	105	42	0	147
SMA type 2	6	97	53	156
SMA type 3	0	6	81	87
Total	111	145	134	390

Data only refers to patients identified by both a neuromuscular center (information about SMA type) and a genetic institute (information about age at genetic diagnosis).
