# Supplementary material for: De-duplicating patient records from three independent data sources reveals the incidence of rare neuromuscular disorders in Germany
Source: Orphanet J Rare Dis. 2019 Jun 24;14:152. doi: 10.1186/s13023-019-1125-2 (PMC6591958; doi:10.1186/s13023-019-1125-2)
Supplement: Supplementary file 3 — Incidences based on the updated natality rate in Germany for DMD and SMA. Table showing all incidences for DMD and SMA based on the updated natality rate in Germany. (PDF 100 kb) [file 13023_2019_1125_MOESM3_ESM.pdf]

Incidences based on the updated natality rate in Germany for spinal muscular atrophy (SMA)

Year of birth	Natality rate in Germany	Distinct patients with SMA	Incidence per 10,000
1995	765221	21	0.27
1996	796013	29	0.36
1997	812173	22	0.27
1998	785034	35	0.45
1999	770744	52	0.67
2000	766999	67	0.87
2001	734475	81	1.10
2002	719250	55	0.76
2003	706721	62	0.88
2004	705622	65	0.92
2005	685795	67	0.98
2006	672724	72	1.07
2007	684862	44	0.64
2008	682514	50	0.73
2009	665126	68	1.02
2010	677947	73	1.08
2011	662685	59	0.89
2012	673544	63	0.94
2013	682069	66	0.97
2014	714927	97	1.36
2015	737575	59	0.80
2016	792121	51	0.64
2017	785000	25	0.32

Incidences based on the updated natality rate of male births in Germany for Duchenne muscular dystrophy (DMD)

Year of birth	Male births in Germany	Distinct patients with DMD	Incidence of DMD per 10,000
1995	392729	75	1.91
1996	409213	68	1.66
1997	417006	76	1.82
1998	402865	83	2.06
1999	396296	84	2.12
2000	393323	77	1.96
2001	377586	97	2.57
2002	369277	68	1.84
2003	362709	74	2.04
2004	362017	82	2.27
2005	351757	77	2.19
2006	345816	85	2.46
2007	351839	73	2.07
2008	349862	57	1.63
2009	341249	63	1.85
2010	347237	65	1.87
2011	339899	57	1.68
2012	345629	52	1.50
2013	349820	43	1.23
2014	366835	43	1,17
2015	378478	19	0.50
2016	405585	9	0.22
2017	402510	6	0.15
