# Supplementary material for: De-duplicating patient records from three independent data sources reveals the incidence of rare neuromuscular disorders in Germany
Source: Orphanet J Rare Dis. 2019 Jun 24;14:152. doi: 10.1186/s13023-019-1125-2 (PMC6591958; doi:10.1186/s13023-019-1125-2)

Distinct patients per 100,000 inhabitants allocated to the first digit of the postal code with dystrophinopathies (A) or SMA (B)

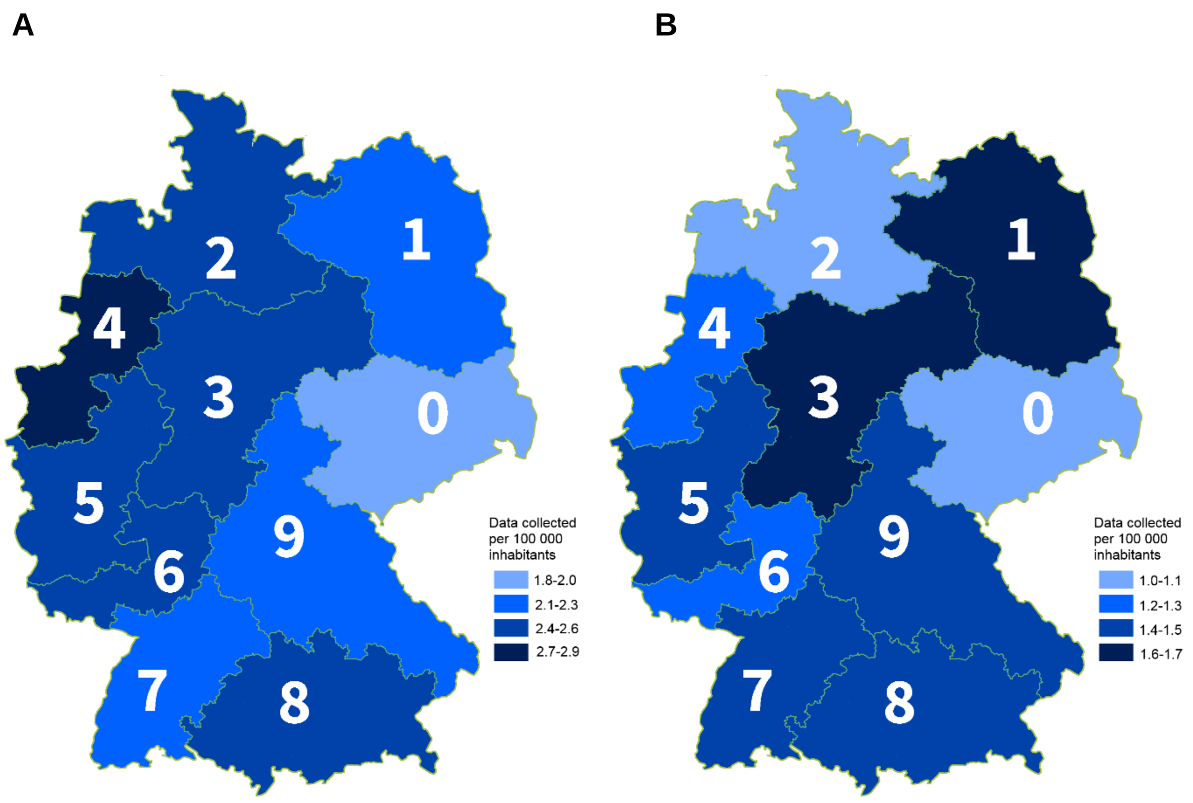

Supplement: Supplementary file 4 — Distinct patients per 100,000 inhabitants allocated to the first digit of the postal code with dystrophinopathies or SMA. Map of Germany presenting data on the regional distribution of patients with dystrophinopathies or SMA. (PDF 240 kb) [file 13023_2019_1125_MOESM4_ESM.pdf]
